# Effect of noise and green space exposure on depression, anxiety and stress among the Lebanese population

**DOI:** 10.1371/journal.pone.0344534

**Published:** 2026-03-20

**Authors:** Raseel Youssef, Nour Dassuki, Dania El Natour, Jana Al Achcar, Rola Maadarani, Ghina Krayker, Bilal Azakir, Jad El Masri, Pascale Salameh

**Affiliations:** 1 Faculty of Medicine, Beirut Arab University, Beirut, Lebanon; 2 Department of Family Medicine, American University of Beirut Medical Center, Beirut, Lebanon; 3 Saint George University of Beirut, Beirut, Lebanon; 4 Department of Anatomy, Cell Biology and Physiological Sciences, Faculty of Medicine, American University of Beirut, Beirut, Lebanon; 5 Faculty of Medical Sciences, Lebanese University, Beirut, Lebanon; 6 Institut National de Santé Publique d’Épidémiologie Clinique et de Toxicologie-Liban (INSPECT-LB), Beirut, Lebanon; 7 Faculty of Pharmacy, Lebanese University, Hadat, Lebanon; 8 Gilbert and Rose-Marie Chagoury School of Medicine, Lebanese American University, Beirut, Lebanon; 9 Department of Primary Care and Population Health, University of Nicosia Medical School, Nicosia, Cyprus; Stanford University School of Medicine, UNITED STATES OF AMERICA

## Abstract

**Background:**

Environmental exposure significantly influences mental well-being. Green spaces offer psychological benefits, while noise exposure is a recognized environmental stressor. However, their effects on mental health outcomes such as depression, anxiety, and stress remain underexplored in Lebanon. This study investigates the influence of green space and noise exposure on mental health in the Lebanese population.

**Methods:**

A cross-sectional study was conducted using an online survey distributed across the Lebanese population. A total of 653 participants aged 18–65 years completed the questionnaire, which assessed socio-demographics, green space exposure, noise exposure, and mental health using the Arabic versions of the validated scales for depression (PHQ-9), anxiety (GAD-7), and stress (PSS-10). Data was analyzed using SPSS version 25.

**Results:**

Higher green space exposure—such as proximity to greenery, views of natural environments, and more frequent visits—was significantly associated with lower levels of depression, anxiety, and stress (p < 0.05). Conversely, greater noise exposure at home or work, and symptoms such as sleep disturbance, irritability, or difficulty concentrating due to noise, were significantly associated with higher scores across all three mental health domains (p < 0.001). Multiple linear regression showed that some noise-related symptoms were consistently associated with higher depression, anxiety, and stress.

**Conclusions:**

Environmental exposures play a critical role in shaping the mental health. Increasing access to green spaces and reducing noise pollution may serve as effective public health interventions to decrease levels of depression, anxiety, and stress among Lebanese residents. Urban planning and public policy should integrate these findings into mental health promotion strategies.

## 1. Introduction

Urbanization is rapidly transforming the world, with over half the population now living in cities, a number expected to grow. While urban life offers several benefits, it also brings environmental challenges that impact health. Among these, two critical yet contrasting urban environmental exposures, green space and noise pollution, have emerged as significant factors in shaping psychological well-being [1]. Green spaces, such as parks, gardens, and urban greenery, have long been studied for their health benefits. Research has linked them to lower risks of cardiovascular disease, sarcopenia, and adverse health outcomes such as stroke, diabetes, and mental health disorders [1–8].

Increasing attention has been given to their impact on mental well-being. Studies indicate that exposure to green spaces improves mood, reduces stress, and lowers the risk of depression and anxiety [4,9–13].

In childhood, proximity to greenery is associated with lower rates of hyperactivity and behavioral issues [14,15], while in adolescence, it correlates with improved emotional resilience and reduced psychological distress [15–17].

Meta-analyses reinforce these findings, showing significant links between green space exposure and lower risks of depression, anxiety, and stress-related disorders [18,19]. Additionally, studies in the US and Europe highlight the protective role of green environments against poor mental health [20,21]. A study in urban Texas further supported this association, finding that neighborhoods with higher Nature Scores had significantly lower mental health utilization rates for anxiety and stress [22]. However, more research is needed on longitudinal changes, particularly in early adolescence [23,24].

The interaction between green space and noise exposure is complex and can have significant implications for mental health outcomes. Green spaces can help reduce stress and anxiety by buffering the negative effects of noise. For instance, studies in Zurich and Shanghai found that vegetation can lessen noise perception and lower psychological stress, though benefits may plateau beyond a certain point [19, 25]. Conversely, noise can also diminish the restorative value of green spaces, as traffic sounds interfere with calming natural sounds like birdsong [26]. Thus, the mental health benefits of green spaces depend on both noise levels and sound quality.

Great attention has been devoted to this matter as noise pollution is regarded as the second greater environmental stressor impacting human health and well-being, after air pollution [27]. Noise exposure has been associated with negative mental health outcomes, including increased stress, anxiety, and depression [28–34].

Adolescents exposed to high noise levels had a 1.56 times greater incidence of depression and 1.60 times higher anxiety levels compared to those in quieter environments [35]. A study conducted in Zurich found that noise pollution was associated with higher levels of perceived stress and long-term physiological stress responses [36]. Similarly, a study in England found that transportation noise was a significant mediator of factors associated with depression prevalence, particularly in areas with higher levels of deprivation [37]. Although several studies and meta-analyses have linked road traffic noise to increased risks of depression and anxiety—with some reporting a 4% rise in depression and a 12% rise in anxiety per 10 dB increase—the overall quality of evidence remains “very low” [29–38]. Despite this, the biological plausibility of the relationship suggests noise should still be considered a potential risk factor for mental health issues [30]. Notably, noise pollution remains an underexplored environmental determinant in mental health research, often overlooked even in broader studies on atmospheric pollution [39].

Despite growing global interest in the mental health impacts of environmental exposures, Lebanon lacks integrated research examining the combined effects of green space and noise pollution on depression, anxiety, and stress. While some studies have explored these factors separately, most of the existing literature in Lebanon lacks the direct assessment of the association between green space and noise exposure and mental health. Therefore, this study aims to assess the effect of green space and noise exposure with depression, anxiety, and stress among the Lebanese population, in addition to assessing their interaction with sociodemographic factors.

## 2. Methodology

### 2.1. Study design and settings

This cross-sectional study targets the Lebanese population, aiming to investigate the effect of green space and noise exposure on mental health parameters: depression, anxiety, and stress. An online questionnaire was developed using Google Forms as a part of the study.

### 2.2. Study participants

A Google Forms link was used to collect data from participants; the link was distributed on several social media platforms using snowball method. Data was collected between May and June 2025. Included responders were Lebanese and aging above 16 years were included in the study, while excluded responders were those not residing in Lebanon, younger than 16 years of age, illiterate, deaf, and those with no access to technology or internet.

### 2.3. Questionnaire

The questionnaire consisted of 6 main sections, and to be noted, all the scales have validated English and Arabic versions.

1. The first section consists of some study variables, including sociodemographic characteristics such as age, gender, work, education, and marital status.
2. Second section assessed depression using the Patient Health Questionnaire-9 (PHQ-9), a 9-item questionnaire used to screen and measure the severity of depression symptoms over the past two weeks. Each item will be rated from 0 to 3, with a total score ranging from 0 to 27 [40].
3. Third section assessed anxiety using the Generalized Anxiety Disorder-7 (GAD-7), a 7-item questionnaire based on DSM-IV criteria used to measure the severity of generalized anxiety disorders. Score will be calculated by assigning scores of 0, 1, 2, and 3 to the response categories, respectively, of “not at all,” “several days,” “more than half the days,” and “nearly every day.” The total score ranges from 0 to 21 [41].
4. Fourth section assessed stress using the Perceived stress scale (PSS-10), a 10-item scale used to assess stress level and evaluate the degree to which an individual perceives their life as unpredictable, uncontrollable, and overwhelming over the past month. Each item was rated from 0 to 4, with a total score ranging from 0 to 40 [42].
5. Fifth section assessed green space exposure, where participants were asked some questions about their place of residency and its surroundings, and the frequency of exposure to green spaces.
6. Sixth section assessed noise exposure, where participants were asked about noise exposure inspired by the 10 items noise exposure questionnaire (NEQ10) described by Johnson et al [43]. This scale was modified carefully to suit the nature of noise that the Lebanese people are exposed to.

### 2.4. Ethical considerations

This study was approved by the institutional review board IRB of Beirut Arab University (ID: 2025-H-0161-M-R0762) on April 28, 2025. The study respected the anonymity of the participants, and all those included signed an online informed consent on the first page of the survey. All participants were also informed about the objectives and benefits of the study before starting, and all patients gave online consent before starting the survey. This study was conducted in accordance with the Declaration of Helsinki [44].

### 2.5. Statistical analysis

The dataset collected underwent cleaning and coding; afterward data were analyzed using SPSS version 25. Descriptive analysis was conducted where continuous variables were expressed as mean ± standard deviation (SD), while categorical variables were presented as numbers and percentages. As for the bivariate analysis, the independent samples t-test was used to test mean differences between two groups and one-way ANOVA was used to compare means between more than two groups. As for the multivariable analysis, multiple linear regressions were conducted to assess the correlation between depression, anxiety, and stress with all factors having p value <0.2 in bivariate analysis. Statistically significant results were considered when p value <0.05 was observed.

## 3. Results

### 3.1. Sociodemographic characteristics

Table 1 shows the sociodemographic characteristics of the included sample. This study recruited a total of 653 participants, the majority of whom are females (62.3%), aged between 18 and 25 years (66.6%), and single (80.2%). As for educational status, 85.3% were university students. Only 17.8% reported working in healthcare sectors, while more than half were unemployed (53.3%).

**Table 1:** Demographic data of respondents (N = 653).

Factor	Category	N (%)
Gender	Male	246 (37.7%)
	Female	407 (62.3%)
Age	<18	41 (6.3%)
	18-25	435 (66.6%)
	26-35	81 (12.4%)
	36-45	58 (8.9%)
	46-64	38 (5.8%)
Work	Health care	116 (17.8%)
	Engineers and technician	42 (6.4%)
	Business and others	147 (22.5%)
	Unemployed	348 (53.3%)
Educational level	Illiterate	16 (2.5%)
	School student	80 (12.3%)
	University	557 (85.3%)
Marital status	Single	524 (80.2%)
	Married	126 (19.3%)
	Divorced	2 (0.3%)
	Widowed	1 (0.2%)

**Fig 1:**
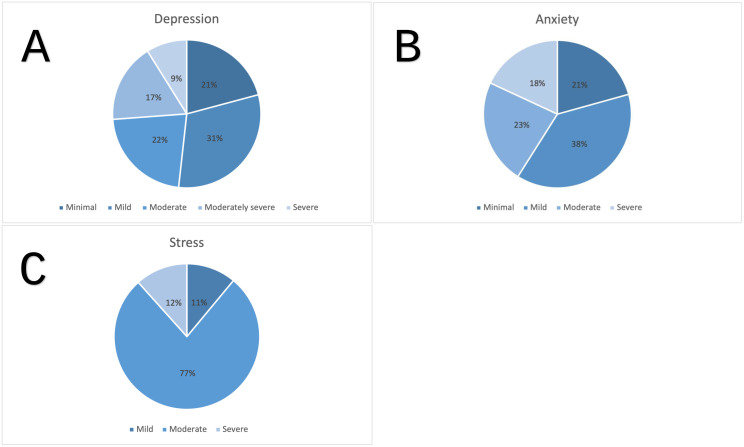
Distribution of depression (A), anxiety (B), and stress (C) severity categories among participants.

Fig 1 shows the distribution of depression, anxiety, and stress severity among participants. For depression, 21% reported minimal symptoms, 31% mild, 22% moderate, 17% moderately severe, and 9% severe symptoms. Regarding anxiety, 21% had minimal symptoms, 38% mild, 23% moderate, and 18% severe anxiety. Stress levels were predominantly moderate (77%), followed by severe (12%) and mild stress (11%).

Table 2 shows the bivariate association between sociodemographic characteristics and depression, anxiety, and stress. Gender was found to be significantly associated with stress levels, with females having higher stress levels than males (20.61 ± 5.46 vs 18.84 ± 5.62) (p < 0.001). Divorced participants had higher levels of depression compared to others (21.00 ± 8.48; p = 0.014). However, no statistically significant associations were reported between the studied mental health parameters and age groups, educational level, or working sector.

**Table 2:** Bivariate association between sociodemographic characteristics and depression, anxiety, and stress.

Factor	Category	Depression		Anxiety		Stress	
		Mean ± SD	P value	Mean ± SD	P value	Mean ± SD	P value
Gender	Male	10.10 ± 6.71	0.339	8.74 ± 6.22	0.084	18.84 ± 5.62	<0.001*
	Female	10.60 ± 6.38		9.57 ± 5.31		20.61 ± 5.46	
Age	<18	11.10 ± 6.26	0.209	10.49 ± 5.92	0.186	21.34 ± 6.07	0.088
	18-25	10.48 ± 6.25		9.13 ± 5.49		19.91 ± 5.37	
	26-35	11.04 ± 7.15		9.58 ± 5.96		20.56 ± 5.70	
	36-45	10.05 ± 7.10		9.95 ± 6.44		19.53 ± 6.46	
	46-64	8.18 ± 7.16		7.68 ± 5.53		18.08 ± 5.46	
Work	Health care	10.55 ± 6.40	586	9.21 ± 5.35	0.516	19.61 ± 5.15	0.06
	Engineers and technician	11.29 ± 5.94		10.36 ± 5.42		21.43 ± 4.19	
	Business and others	9.86 ± 6.86		8.87 ± 6.16		19.12 ± 5.69	
	Unemployed	10.50 ± 6.47		9.30 ± 5.61		20.22 ± 5.78	
Educational level	Illiterate	9.88 ± 9.45	0.103	9.00 ± 7.64	0.118	19.81 ± 6.51	0.352
	School student	11.86 ± 6.92		10.49 ± 6.17		20.79 ± 5.46	
	University	10.22 ± 6.33		9.09 ± 5.53		19.82 ± 5.57	
Marital status	Single	10.64 ± 6.45	0.014*	9.33 ± 5.66	0.26	20.09 ± 5.56	0.496
	Married	9.40 ± 6.52		8.80 ± 5.73		19.33 ± 5.71	
	Divorced	21.00 ± 8.48		15.5 ± 4.95		22.00 ± 2.82	

### 3.2. Association between green space exposure and depression, anxiety, and stress

Table 3 shows the bivariate association between green space exposure and depression, anxiety, and stress. Those residing in urban areas were shown to have higher anxiety levels than those in rural areas (9.63 ± 5.893 vs 8.73 ± 5.343; p = 0.04), and surprisingly, those living in apartments scored a lower stress level than those living in a house (19.13 ± 5.30 vs 20.55 ± 5.71; p = 0.001). Home and work view was significantly associated with depression, anxiety and stress, with those viewing green spaces from their homes scoring less depression level (p = 0.009), less anxiety (p = 0.031), and less stress (p = 0.005), and those viewing green spaces from work/study scoring less depression level (p = 0.037) and less stress (p = 0.016).

**Table 3:** Bivariate association between green space exposure and depression, anxiety, and stress.

Factor	Category	Depression		Anxiety		Stress	
		Mean ± SD	P value	Mean ± SD	P value	Mean ± SD	P value
Place of residency	Urban	10.67 ± 6.590	0.227	9.63 ± 5.893	0.044*	20.24 ± 5.591	0.107
	Rural	10.05 ± 6.395		8.73 ± 5.343		19.52 ± 5.563	
Work/study area	Urban	10.3 ± 6.32	0.419	9.34 ± 5.74	0.543	20.12 ± 5.62	0.164
	Rural	10.77 ± 7.06		9.02 ± 5.5		19.42 ± 5.46	
Home condition	House	10.63 ± 6.48	0.329	9.54 ± 5.62	0.146	20.55 ± 5.71	0.001*
	Apartment	10.13 ± 6.54		8.88 ± 5.75		19.13 ± 5.30	
Have a private garden	Yes	10.46 ± 6.61	0.882	9.00 ± 5.48	0.369	19.55 ± 5.64	0.168
	No	10.39 ± 6.35		9.41 ± 5.80		20.17 ± 5.64	
View from home	Buildings	10.68 ± 6.44	0.161	9.52 ± 5.66	0.11	20.27 ± 5.64	0.041*
	Green spaces	9.82 ± 6.32	0.009*	8.82 ± 5.45	0.031*	19.39 ± 5.45	0.005*
	Water body	10.25 ± 6.34	0.82	8.62 ± 5.55	0.328	18.97 ± 5.81	0.127
	Factories	10.85 ± 6.694	0.81	8.31 ± 3.72	0.376	19.85 ± 7.55	0.95
	Highway	10.83 ± 673	0.47	9.92 ± 6.08	0.19	19.50 ± 5.73	0.366
	Sports arena, concert venue	9.73 ± 7.126	0.615	10.14 ± 5.89	0.461	20.45 ± 6.061	0.662
View from work/study place	Buildings	10.32 ± 6.37	0.496	9.31 ± 5.636	0.652	20.12 ± 5.61	0.146
	Green spaces	9.81 ± 6.58	0.037*	8.94 ± 5.67	0.209	19.35 ± 5.606	0.016*
	Waterbody	9.01 ± 6.408	0.061	8.93 ± 5.92	0.613	18.41 ± 5.23	0.017*
	Factories	10.33 ± 5.909	0.944	10.40 ± 6.306	0.26	18.57 ± 5.36	0.168*
	Highway	10.15 ± 6.36	0.603	9.20 ± 5.82	0.905	19.14 ± 5.76	0.066
	Sports arena, concert venue	9.11 ± 6.67	0.164	8.56 ± 6.070	0.391	18.42 ± 6.53	0.059
Distance from nearest green space from home	<5 minutes	9.85 ± 6.51	0.045*	8.83 ± 5.57	0.041*	19.48 ± 5.45	0.026*
	5-15 minutes	10.46 ± 5.93		9.19 ± 5.68		19.95 ± 5.45	
	15-30 minutes	11.44 ± 6.87		9.75 ± 5.71		20.44 ± 5.29	
	>30 minutes	11.82 ± 7.05		10.84 ± 5.97		21.55 ± 6.47	
Frequency of green space visit	Daily	9.26 ± 6.75	0.153	8.42 ± 5.89	0.086	18.58 ± 5.32	0.023*
	Few times a week	9.66 ± 6.87		8.28 ± 5.43		19.21 ± 5.80	
	Weekly	10.46 ± 6.33		9.37 ± 5.38		20.11 ± 5.92	
	Few times a month	10.78 ± 6.21		9.67 ± 5.47		20.18 ± 5.19	
	Rarely/never	11.08 ± 6.49		9.82 ± 6.03		20.77 ± 5.69	
Average time spent per visit	< 15 minutes	10.79 ± 6.20	0.711	9.88 ± 5.71	0.343	20.21 ± 5.31	0.449
	15-30 minutes	9.93 ± 6.65		9.20 ± 5.36		19.80 ± 4.68	
	30-60 minutes	9.86 ± 5.89		8.81 ± 5.08		19.68 ± 5.44	
	>1 hour	10.34 ± 7.21		8.55 ± 6.05		19.02 ± 6.51	
Visit usually	Alone	9.80 ± 6.22	0.502	8.93 ± 562	0.801	19.10 ± 4.85	0.254
	With a company	10.28 ± 6.59		9.09 ± 5.52		19.80 ± 5.69	
Weekend usually spent in	City	10.76 ± 6.47	0.301	9.85 ± 5.89	0.015*	20.43 ± 5.66	0.052
	Mountains	9.93 ± 6.51		8.50 ± 5.28		19.47 ± 5.48	
	By the sea, lake or river	10.56 ± 6.78		9.19 ± 5.99		19.00 ± 5.30	
Summer vacation spent in	City	11.23 ± 6.53	0.072	10.31 ± 6.00	<0.001*	20.70 ± 5.4	0.034*
	Mountains	9.74 ± 6.36		8.30 ± 5.20		19.25 ± 5.68	
	By the sea, lake or river	9.74 ± 6.47		8.58 ± 5.38		19.64 ± 5.56	

Adding to that, proximity to green space was a significant factor; being 5 minutes away from green space was associated with lower depression levels (p = 0.045), anxiety (p = 0.041), and stress (p = 0.026) in comparison to those living in farther areas. Similarly, spending weekends or summer vacations in the mountains was associated with lower anxiety and stress in comparison to spending them in the city (p < 0.05). Finally, those who visit green spaces daily, was shown to have less stress level than others (p = 0.023).

### 3.3. Association between green space exposure and depression, anxiety, and stress

Table 4 shows the bivariate association between noise exposure and depression, anxiety, and stress. Higher levels of anxiety, stress, and depression were significantly associated with frequent or constant exposure to loud noises at home and work, inability to concentrate or sleep, headaches/ irritability due to loud noise, and needing to use noise-reducing measures (e.g., earplugs) with a p-value <0.001 for all.

**Table 4:** Bivariate association between noise exposure and depression, anxiety, and stress.

Factor	Category	Depression		Anxiety		Stress	
		Mean ± SD	P value	Mean ± SD	P value	Mean ± SD	P value
Exposed to loud noise at home	Always	11.85 ± 7.13	< 0.001*	10.89 ± 6.28	<0.001*	21.01 ± 5.05	<0.001*
	Frequently	11.28 ± 6.03		9.77 ± 5.34		20.45 ± 5.41	
	Rarely	9.19 ± 6.27		8.31 ± 5.38		19.28 ± 5.76	
	Never	6.73 ± 6.79		5.70 ± 5.58		16.55 ± 5.90	
Exposed to loud sounds while working on a paid job	Always	11.78 ± 7.08	<0.001*	10.93 ± 6.25	<0.001*	21.20 ± 6.01	<0.001*
	Frequently	11.05 ± 6.26		9.54 ± 5.40		20.41 ± 5.27	
	Rarely	9.12 ± 6.12		8.18 ± 5.33		18.97 ± 5.65	
	Never	8.86 ± 6.88		8.00 ± 6.07		18.26 ± 4.91	
Unable to concentrate due to loud sounds	Always	12.35 ± 7.13	<0.001*	11.21 ± 6.20	<0.001*	20.41 ± 5.27	0.003*
	Frequently	11.19 ± 5.89		9.50 ± 5.25		20.72 ± 4.99	
	Rarely	8.86 ± 6.33		8.25 ± 5.46		18.98 ± 6.20	
	Never	7.76 ± 6.69		6.82 ± 5.99		18.82 ± 5.62	
Unable to sleep due to loud sounds	Always	11.81 ± 7.17	<0.001*	11.29 ± 5.99	<0.001*	20.25 ± 5.44	0.021*
	Frequently	11.96 ± 6.41		9.85 ± 5.39		20.70 ± 5.17	
	Rarely	9.96 ± 6.31		8.99 ± 5.61		19.89 ± 5.75	
	Never	7.85 ± 5.64		7.18 ± 5.37		18.54 ± 5.69	
Experiencing headache and/or irritability due to loud noise	Always	13.09 ± 6.79	<0.001*	12.22 ± 5.64	< 0.001*	20.99 ± 5.21	<0.001*
	Frequently	11.70 ± 6.10		10.12 ± 4.97		21.05 ± 4.99	
	Rarely	9.20 ± 6.16		8.13 ± 5.60		19.22 ± 5.78	
	Never	7.42 ± 6.10		6.55 ± 5.43		18.11 ± 6.03	
Having to take action to reduce your noise exposure (earplugs, noise canceling headphones...)	Always	13.12 ± 7.14	<0.001*	11.78 ± 5.98	<0.001*	21.59 ± 5.29	<0.001*
	Frequently	11.30 ± 5.96		9.94 ± 5.25		20.56 ± 4.80	
	Rarely	9.97 ± 6.28		9.01 ± 5.44		19.66 ± 5.78	
	Never	8.80 ± 6.36		7.65 ± 5.63		18.91 ± 5.84	
Noise exposure from	Loud neighbors	10.64 ± 6.47	0.608	9.95 ± 5.56	0.066	20.15 ± 5.46	0.578
	Construction sites	11.04 ± 7.332	0.19	9.62 ± 6.404	0.3	20.38 ± 5.949	0.268
	Children/people on the street	8.93 ± 6.51	0.225	8.07 ± 6.40	0.27	18.70 ± 5.518	0.24
	Firearms (rihes, pistols, shotguns…)	15.02 ± 3.89	0.099	9.60 ± 2.88	0.892	17.00 ± 2.915	0.237
	Industrial activities factories	11.68 ± 7.108	0.246	9.74 ± 5.51	0.615	20.50 ± 5.36	0.55
	Road traffic	10.52 ± 6.48	0.572	9.38 ± 5.69	0.418	20.11 ± 5.47	0.287
	Sport events/ loud music	11.04 ± 8.27	0.613	10.04 ± 6.35	0.467	20.03 ± 4.23	0.737
Time exposed to noise	Morning	9.97 ± 7.03	0.409	8.88 ± 5.80	0.428	19.39 ± 5.07	0.236
	Afternoon	9.83 ± 6.26	0.081	8.63 ± 5.38	0.028*	19.22 ± 5.33	0.012*
	Evening	11.49 ± 6.76	0.057	10.05 ± 5.85	0.105	20.44 ± 5.07	0.301
	Night	11.59 ± 6.45	0.098	10.01 ± 5.56	0.225	20.35 ± 4.58	0.503

None of the noise sources was associated with anxiety, stress, or depression, whereas time of exposure was, with those exposed to noise in the afternoon reporting higher levels of anxiety (p = 0.028) and stress (p = 0.012).

Table 5 shows the multiple linear regression considering depression as a dependent variable. Experiencing headache and/or irritability due to loud noise was significantly associated with depression symptoms (B = 1.14, 95% CI: 0.42–1.85, p = 0.002).

**Table 5:** Multiple Linear Regression Analysis of Environmental and Noise Exposure Factors Associated with Depression (PHQ-9).

	Unstandardized Coefficients		Standardized Coefficients	t	Sig.	95.0% Confidence Interval for B	
	B	Std. Error	Beta			Lower Bound	Upper Bound
(Constant)	7.89	1.901		4.151	<.001	4.157	11.623
Buildings view from home	−0.471	0.617	−0.035	−0.764	0.445	−1.682	0.74
Green spaces view from home	−0.177	0.627	−0.014	−0.282	0.778	−1.408	1.054
Distance of green space from your home	−0.019	0.29	−0.003	−0.065	0.949	−0.589	0.551
Frequency of green space visit	0.304	0.198	0.065	1.534	0.126	−0.085	0.692
Where do you spend most of your summer vacation	−0.528	0.344	−0.06	−1.536	0.125	−1.202	0.147
Level of exposure to loud noise at home	0.567	0.354	0.072	1.603	0.109	−0.127	1.261
Level of exposure to loud sounds while working on a paid job	0.422	0.317	0.056	1.333	0.183	−0.2	1.044
Frequency of not being able to concentrate due to loud sounds	0.199	0.411	0.026	0.485	0.628	−0.607	1.005
Frequency of not being able to sleep due to loud sounds	0.092	0.361	0.013	0.254	0.800	−0.618	0.801
Frequency of experiencing headache and/or irritability due to loud noise	1.136	0.365	0.16	3.11	0.002	0.419	1.854
Frequency of having to take action to reduce your noise exposure	0.563	0.304	0.088	1.851	0.065	−0.034	1.159
Mostly exposed to noise on afternoons	−0.117	0.529	−0.009	−0.221	0.825	−1.156	0.922
Education level (Reference: uneducated)	−0.564	0.577	−0.038	−0.978	0.329	−1.697	0.569
Highway view from home	−0.02	0.684	−0.001	−0.03	0.976	−1.363	1.323
Mostly exposed to noise on evening	0.836	0.686	0.048	1.219	0.223	−0.511	2.184
Mostly exposed to noise on night	1.017	0.81	0.049	1.255	0.210	−0.575	2.608
Marital status: (reference: single)	−0.668	0.604	−0.043	−1.107	0.269	−1.854	0.517
Green space view from work/study	−0.26	0.52	−0.02	−0.5	0.617	−1.28	0.76
Water view from work/study	−0.974	0.824	−0.046	−1.182	0.237	−2.593	0.644
Sports view from work/study	−1.375	1.021	−0.053	−1.347	0.179	−3.379	0.63
Noise source: Construction sites	0.99	0.822	0.048	1.203	0.229	−0.625	2.605
Noise source: Firearms	4.096	2.868	0.055	1.428	0.154	−1.536	9.728

Dependent Variable: PHQ9.

Table 6 shows the multiple linear regression considering anxiety as a dependent variable. Spending summer vacation outside the city (B = –0.76, 95% CI: –1.37 to –0.14, p = 0.016) was significantly associated with lower anxiety scores. In contrast, frequency of experiencing headache and/or irritability due to loud noise (B = 1.36, 95% CI: 0.72–1.99, p < 0.001) and frequency of having to take action to reduce noise exposure (B = 0.62, 95% CI: 0.10–1.15, p = 0.020) were significantly associated with higher anxiety scores.

**Table 6:** Multiple linear regression analysis of environmental and noise exposure factors associated with anxiety (Gad-7).

	Unstandardized Coefficients		Standardized Coefficients	t	Sig.	95.0% Confidence Interval for B	
	B	Std. Error	Beta			Lower Bound	Upper Bound
(Constant)	6.113	1.985		3.08	0.002	2.215	10.012
Females	0.42	0.449	0.036	0.936	0.35	−0.462	1.302
Age	0.043	0.242	0.007	0.176	0.86	−0.432	0.517
Live in rural area	0.017	0.547	0.001	0.031	0.975	−1.058	1.092
Work/study in rural area	−0.023	0.516	−0.002	−0.044	0.965	−1.036	0.991
Buildings view from home	−0.346	0.557	−0.029	−0.621	0.535	−1.441	0.748
Green spaces view from home	0.063	0.558	0.006	0.113	0.91	−1.033	1.16
Distance of green space from your home	0.006	0.255	0.001	0.024	0.981	−0.495	0.507
Frequency of green space visit	0.287	0.178	0.07	1.615	0.107	−0.062	0.636
Spend weekends (Reference: city)	−0.09	0.385	−0.01	−0.232	0.816	−0.846	0.667
Spend summer vacation (Reference: city)	−0.755	0.312	−0.099	−2.417	0.016	−1.369	−0.141
Level of exposure to loud noise at home	0.533	0.309	0.077	1.724	0.085	−0.074	1.14
Level of exposure to loud sounds while working on a paid job	0.454	0.272	0.068	1.672	0.095	−0.079	0.988
Frequency of not being able to concentrate due to loud sounds	−0.152	0.361	−0.022	−0.421	0.674	−0.86	0.556
Frequency of not being able to sleep due to loud sounds	−0.103	0.314	−0.016	−0.328	0.743	−0.719	0.513
Frequency of experiencing headache and/or irritability due to loud noise	1.356	0.323	0.218	4.202	<0.001	0.722	1.99
Frequency of having to take action to reduce your noise exposure	0.623	0.266	0.111	2.341	0.02	0.1	1.145
Mostly exposed to noise on afternoons	−0.235	0.453	−0.02	−0.519	0.604	−1.124	0.654
Education level (Reference: uneducated)	−0.563	0.509	−0.043	−1.106	0.269	−1.563	0.436
Highway view from home	0.366	0.588	0.024	0.623	0.534	−0.788	1.52
Noise source: Loud neighbors	0.762	0.532	0.058	1.431	0.153	−0.284	1.808
Mostly exposed to noise on evenings	0.549	0.606	0.036	0.907	0.365	−0.64	1.739
Mostly exposed to noise on nights	0.446	0.723	0.025	0.617	0.538	−0.974	1.865

Dependent Variable: DAD-7.

Table 7 shows the multiple linear regression considering stress as a dependent variable. Being female (B = 1.22, 95% CI: 0.33–2.12, p = 0.007) was significantly associated with higher stress scores. In contrast, living in a house (B = –1.34, 95% CI: –2.45 to –0.23, p = 0.018) and having a highway view from the workplace (B = –1.12, 95% CI: –2.21 to –0.04, p = 0.042) were significantly associated with lower stress scores. Additionally, higher exposure to loud sounds while working in a paid job was significantly related to increased stress (B = 0.75, 95% CI: 0.20–1.30, p = 0.008). Other environmental and noise-related variables, including private garden access, frequency of green space visits, and noise-related irritability, showed only borderline associations, while the remaining predictors were not significantly related to PSS-10 scores.

**Table 7:** Multiple linear regression analysis of environmental and noise exposure factors associated with stress (PSS10).

	Unstandardized Coefficients		Standardized Coefficients	t	Sig.	95.0% Confidence Interval for B	
	B	Std. Error	Beta			Lower Bound	Upper Bound
(Constant)	18.453	2.028		9.098	<.001	14.47	22.436
Females	1.224	0.454	0.106	2.698	0.007	0.333	2.115
Age	−0.315	0.246	−0.053	−1.277	0.202	−0.798	0.169
Specify your occupation:	0.118	0.193	0.024	0.611	0.542	−0.262	0.498
Live in rural area	0.571	0.601	0.05	0.95	0.342	−0.609	1.751
Work/study in rural area	0.148	0.635	0.012	0.234	0.815	−1.098	1.395
Living in a house	−1.342	0.564	−0.119	−2.378	0.018	−2.45	−0.234
Having a private garden	1.095	0.597	0.095	1.834	0.067	−0.077	2.267
Buildings view from home	−0.293	0.586	−0.025	−0.5	0.617	−1.443	0.858
Green spaces view from home	−0.51	0.584	−0.046	−0.873	0.383	−1.657	0.638
Water view from home	−0.869	0.74	−0.048	−1.174	0.241	−2.323	0.585
Buildings view from work/study	−0.037	0.612	−0.003	−0.061	0.951	−1.238	1.164
Green space view from work/study	−0.478	0.489	−0.043	−0.976	0.329	−1.439	0.483
Highway view from work/study	−1.124	0.551	−0.081	−2.039	0.042	−2.206	−0.042
Water view from work/study	−0.705	0.742	−0.039	−0.95	0.342	−2.161	0.752
Sports view from work/study	−0.571	0.882	−0.026	−0.648	0.517	−2.303	1.16
Factories view from work/study	−0.642	1.083	−0.024	−0.592	0.554	−2.769	1.486
Distance of green space from your home	0.078	0.259	0.014	0.301	0.764	−0.43	0.586
Frequency of green space visit	0.302	0.18	0.075	1.683	0.093	−0.05	0.655
Spend weekends (Reference: city)	−0.108	0.386	−0.012	−0.28	0.78	−0.865	0.65
Spend summer vacation (Reference: city)	−0.344	0.313	−0.046	−1.099	0.272	−0.958	0.271
Level of exposure to loud noise at home	0.489	0.311	0.072	1.574	0.116	−0.121	1.099
Level of exposure to loud sounds while working on a paid job	0.75	0.282	0.115	2.659	0.008	0.196	1.304
Frequency of not being able to concentrate due to loud sounds	−0.407	0.362	−0.061	−1.124	0.261	−1.119	0.304
Frequency of not being able to sleep due to loud sounds	−0.194	0.315	−0.032	−0.617	0.538	−0.813	0.424
Frequency of experiencing headache and/or irritability due to loud noise	0.615	0.323	0.101	1.906	0.057	−0.019	1.249
Frequency of having to take action to reduce your noise exposure	0.427	0.264	0.077	1.616	0.107	−0.092	0.945
Mostly exposed to noise on afternoons	−0.631	0.452	−0.055	−1.397	0.163	−1.518	0.256

Dependent Variable: PSS10.

## 4. Discussion

This study aimed to investigate the relationship between environmental exposures—namely green spaces and noise pollution—and mental health outcomes in the Lebanese population. The results showed lower stress, anxiety and depression levels among frequent green space visitors and higher levels among people exposed to noise pollution both at home and in the study/workplace. These findings align with international literature suggesting that environmental surroundings are closely linked to psychological well-being.

### 4.1. Green space exposure and its association with mental health

Participants living in urban areas reported significantly higher anxiety levels than those in rural settings. On the other hand, those who were living in rural areas showed lower anxiety. This aligns with existing evidence from meta-analyses, systematic reviews, and longitudinal studies, which have demonstrated similar associations, even when the observed effects were small or statistically non-significant [18,45–50].

Our results align with findings from several other studies including reviews that concluded that green space access is associated with better mental health outcomes [10,18,51–53]. Their findings support theories such as the Attention Restoration Theory and Stress Reduction Theory, which propose that simply viewing natural environments can improve mental health by reducing cognitive fatigue and physiological stress responses.

Recent research highlights the “20-minute park” effect, which suggests that even short visits to green spaces can significantly reduce cortisol levels and boost endorphin production. This physiological response contributes to lower stress and greater emotional balance, reinforcing the mental health benefits of brief but regular exposure to natural environments [54].

In the context of the Lebanese population who suffer ongoing socio-economic and urban challenges and especially for those who lives in the capital Beirut, where there is scarcity of green spaces, our study agrees with the growing body of literature highlighting the mental health benefits of exposure to green spaces. Most of the population living in Beirut, who suffer from financial hardship, suffer also from depression, anxiety, and stress. Raad et al also showed a moderate positive correlation (Spearman’s rho = 0.30) between the quality of public spaces and improved psychological well-being, highlighting the importance of spatial quality in urban mental health in a study targeting as well the Lebanese population [55].

Moreover, evidence suggests that increasing green space and reducing noise exposure can reduce psychological distress and even lower the risk of ischemic stroke in the Lebanese population [56]. Additionally, urban–rural comparisons have shown that while urban dwellers may experience more persistent depression, rural populations report higher levels of sadness and loneliness, pointing to the importance of public space interventions in both settings [57]. Our study shows that there is an association between green space access and better mental health, and this underscores the urgent need for public policies to enhance and protect urban greenery, especially in under-resourced communities.

In cities like Beirut, improving the availability, safety, and quality of green spaces could serve as a low-cost, high-impact approach to support mental resilience in the face of overlapping economic, environmental, and public health crises.

### 4.2. Noise exposure and its association with mental health

Noise pollution is increasingly recognized as the second most significant environmental stressor affecting human health after air pollution [27]. Chronic noise exposure has been linked to higher risks of depression, anxiety, and stress in multiple contexts. For example, a meta-analysis by Dzhambov and Lercher (2019) concluded that road traffic noise significantly increases the risk of depression and anxiety disorders [29]. Similarly, Gong et al. (2022) found that annoyance from environmental noise is linked to depression, generalized anxiety disorders, and poorer general mental health [28]. Moreover, adolescents in noisy urban environments have been shown to experience a 1.5-fold higher incidence of depression and anxiety compared to peers in quieter areas [35].

In our study, the timing of noise also appeared relevant, with afternoon exposure significantly associated with elevated anxiety and stress levels. This aligns with research suggesting that disruption of circadian rhythms and concentration during daytime activities may exacerbate the psychological burden of noise [36]. Physiologically, prolonged exposure to high noise levels has been shown to activate the hypothalamic–pituitary–adrenal (HPA) axis, increase cortisol secretion, and elevate blood pressure, mechanisms that may underlie the observed increase in psychological distress [30].

Lebanon’s unique urban context may intensify these effects. Prior studies in Beirut documented how persistent environmental noise contributes to stress, sleep disturbances, and hearing problems [58]. More recently, El Masri et al. (2024) demonstrated that the combined exposure to noise and lack of green space significantly increased the risk of ischemic stroke in Lebanon, highlighting the intersection between environmental exposure and health risks [56].

### 4.3. Limitations

Several limitations in the study should be acknowledged. First, the method applied is cross-sectional study which means data collection was done at a single time point. This may hinder the ability to establish a correlation between noise or green space and psychological well-being. Second, the results were collected by a questionnaire to report environmental exposure and mental health symptoms, which may be subjected to recall, selection, information and residual confounding biases. Third, the assessment of exposure was relatively subjective taking into consideration the geographic information system, the green space mapping and the noise measurement. These factors may have affected the accuracy of the results. Fourth, even though a large sample was taken for this study, it may not be fully representative of the entire Lebanese population in terms of geographic distribution and socioeconomic class, which may hinder generalization. Fifth, extraneous variables may have affected the results, such as pollution, habits, lifestyle and many other factors. Finally, in the last several years Lebanon was facing tremendous economic and political problem, which may had independently affected the Lebanese population mental health and well-being.

## 5. Conclusion

In conclusion, this study highlights the significant impact of green space access and noise pollution on mental health. The results underscore the urgent need for urban planning strategies, given Lebanon’s dense urbanization, and public health interventions that prioritize environmental quality especially in areas of poor living conditions. Creating and providing access to more green spaces like parks and natural spaces and taking measures to reduce noise pollution are essential steps that should be taken to improve the well-being of the Lebanese population who are experiencing growing rates of mental health disorders. Future studies using longitudinal designs and objective environmental exposure measures are needed to better assess the effect of green space and noise exposure on mental health.

## Supporting information

S1 File Data. (XLSX)
